# The active metabolite of Epimedii Folium promotes hippocampal neurogenesis in APP/PS1 mice by alleviating mitochondrial dysfunction

**DOI:** 10.3389/fphar.2025.1546256

**Published:** 2025-04-25

**Authors:** Jia-Ming Bai, Tong Li, Xue Di, Jing-Xian Yang, Zhao-Qi Cui, Dong-Yu Min, Yu-Feng Shen, Si-Yu Shan, Ye-Xin Zhang, Yi-Jun Shi, Zhi-Li Xu, De-Qiang Dou, Hong-He Xiao

**Affiliations:** ^1^ School of Pharmacy, Liaoning University of Traditional Chinese Medicine, Dalian, China; ^2^ Traditional Chinese Medicine Experimental Center, Affiliated Hospital of Liaoning University of Traditional Chinese Medicine, Shenyang, China

**Keywords:** Alzheimer’s disease, Epimedii folium, neural stem cell, hippocampal neurogenesis, mitochondria

## Abstract

**Introduction:**

Alzheimer’s disease (AD), the most common form of dementia, currently has no effective cure. Epimedii Folium (EF), a traditional Chinese medicine known as Yin-yang-huo, has demonstrated significant neuroprotective properties.

**Methods:**

In this study, neural stem cells overexpressing the APPswe gene (APP-NSCs) were used as an *in vitro* AD model. The CCK-8, LDH, neurosphere formation, and BrdU incorporation assays were employed to identify the most effective bioactive metabolite of EF in promoting NSC proliferation. Subsequently, JC-1 staining, ATP quantification, and ROS assays were conducted to evaluate the protective effects of Icariside II (ICS II)-identified as the most effective metabolite-on mitochondrial function. APP/PS1 transgenic mice received an oral administration of 10 mg/kg ICS II for 7 weeks. Cognitive function was assessed using the Morris water maze and nest-building tests, while H&E and Nissl staining were used to evaluate brain tissue pathology. Transmission electron microscopy (TEM) examined the ultrastructural integrity of hippocampal neurons, immunofluorescence assessed hippocampal neurogenesis, and Western blotting quantified proteins involved in mitochondrial dynamics. Additionally, Rotenone (Rot), a mitochondrial respiratory chain inhibitor, was applied to disrupt mitochondrial function, allowing an evaluation of whether the neurogenesis-promoting effect of ICS II depends on maintaining mitochondrial structure and function.

**Results and discussion:**

The results demonstrated that ICS II exhibited the strongest capacity to promote APP-NSC proliferation (P < 0.01, η^2^ = 0.845), followed by Icariin and Icaritin. ICS II treatment significantly ameliorated cognitive deficits (*P* < 0.01, η^2^ = 0.883), neuronal damage, and impairments in neurogenesis in adult APP/PS1 mice. Moreover, ICS II rescued mitochondrial damage by upregulating fusion proteins (Mfn1 and Mfn2) and downregulating fission proteins (p-Drp1/Drp1 and Mff); however, these protective effects were negated by Rot administration. In conclusion, this study identifies ICS II as one of the most effective metabolites of EF, promoting hippocampal neurogenesis and alleviating mitochondrial dysfunction in APP/PS1 mice, thereby offering promising therapeutic potential for AD.

## 1 Introduction

Alzheimer’s disease (AD), also referred to as senile dementia, is the most prevalent form of dementia and significantly impacts the physical health and quality of life of elderly individuals. The pathogenesis of AD is complex and remains incompletely understood. Key hypotheses include the classic mechanisms of amyloid-beta (Aβ) deposition and tau protein hyperphosphorylation. As research progresses, additional contributing factors have been identified, such as neuroinflammation, oxidative stress, cholinergic deficits, excitotoxicity, mitochondrial dysfunction, genetic mutations, prion infections, gut microbiota dysbiosis, and impaired neurogenesis (Golde et al., 2018; Jia et al., 2020). Notably, neuronal degeneration and loss are common outcomes of these pathogenic factors, underscoring the critical importance of repairing damaged neurons as a therapeutic strategy for AD.

Under physiological conditions, neural stem cells (NSCs) located in the subgranular zone (SGZ) of the hippocampal dentate gyrus (DG) proliferate and differentiate to generate new neurons. These new neurons establish connections with pre-existing neural networks by projecting axons and dendrites to the CA1 and CA3 regions of the hippocampus, thereby replenishing and repairing damaged neurons and synaptic connections. This process, known as hippocampal neurogenesis, is crucial for maintaining synaptic plasticity and the structural and functional integrity of the hippocampus (Doetsch et al., 1999). Unfortunately, pathological factors such as Aβ plaques, neuroinflammation, and oxidative stress impair the neurogenesis mechanism in the brain of AD patient. As a result, the proliferation and differentiation of NSCs are significantly reduced, and newly generated intermediate neurons struggle to mature into fully functional neurons (Christie and Turnley, 2013; Mao et al., 2015; MS and JWinpenny, 2009; Waldau and Shetty, 2008). Hippocampal neurogenesis is impaired in AD patient, with the number of newly generated neurons being less than one-third of the normal level. Moreover, the number of new neurons continues to decline as the disease progresses (Choi and Tanzi, 2019; Moreno-Jiménez et al., 2019). In AD model mice, such as APP/PS1 and 3xTg mice, the number of NSCs and newly generated neurons in the hippocampus is significantly lower compared to age-matched wild-type mice. Conditional ablation of hippocampal neurogenesis further exacerbates cognitive impairments (Hollands et al., 2017; Mireille et al., 2019). Fortunately, small molecule compounds such as metformin (Fatt et al., 2015) and ethosuximide (Shashi Kant et al., 2015) have been shown to promote hippocampal neurogenesis, increase the number of hippocampal neurons, and restore cognitive function in AD model animals. Thus, restoring neurogenesis in the brain represents a promising strategy for treating AD. Promoting endogenous neuro-regeneration may offer an effective therapeutic approach for AD and other neurodegenerative diseases.

Contemporary ethnopharmacological investigations have identified multiple phytocompounds from ancestral pharmacopeias that demonstrate multimodal neuroprotection in AD pathology (Dey et al., 2017; Sharma et al., 2024; Siddiqui et al., 2024; Tripathi et al., 2024). Epimedii Folium (EF) is a widely used kidney-tonifying traditional Chinese medicine known for its effects in tonifying kidney-yang, strengthening bones and tendons, and dispelling wind-dampness. EF primarily contains flavonol glycosides with an eight-isopentenyl structure, including Icariin (ICA), Epimedin A, Epimedin B, Epimedin C, Icariside I (ICS I), Icariside II (ICS II), Icaritin, and Anhydroicaritin. According to the 2020 edition of the Pharmacopoeia of the People’s Republic of China, Epimedin A, Epimedin B, Epimedin C, and ICA are quality control markers for EF, while Epimedin A, Epimedin B, Epimedin C, ICA, and ICS II are quality control markers for processed EF (Committee, 2020). Numerous studies have demonstrated that EF and its active metabolites can improve neural damages, enhance cognitive function in animal models, and exhibit multiple neuroprotective effects by modulating neuroinflammation, reducing oxidative stress, and increasing neurotrophic factors (Chen et al., 2016; Cho et al., 2017; Li et al., 2015; Wang et al., 2023). For example, ICA and Icaritin have been reported to protect against neuroinflammation in depressive mice through regulation of high mobility group protein (HMGB1) and NF-κB pathways (Liu et al., 2019). Epimedin B has been reported to protect against 1-methyl-4-phenyl-1,2,3,6-tetrahydropyridin (MPTP)-induced PD mice through regulation of apoptosis and endoplasmic reticulum stress (Zhang M. et al., 2022). In our previous study, we found that ICS II could promote hippocampal neurogenesis *via* activation of Wnt/β-catenin signaling pathway to improve the cognitive function of APP/PS1 mice (Xiao et al., 2022; Xiao et al., 2021). However, the comparative efficacy of these metabolites in promoting NSC proliferation and enhancing hippocampal neurogenesis specifically in AD models remains to be systematically investigated. Particularly, the identification of the most potent metabolite for neurogenic enhancement in AD pathogenesis has not been established. Furthermore, while emerging evidence implicates mitochondrial dynamics as a critical regulator of NSC fate determination, the potential mitochondrial-mediated mechanisms underlying EF metabolite-induced neurogenesis remain unexplored.

In this study, by using neural stem cells overexpressing the APPswe mutant amyloid precursor protein (APP-NSCs) as an AD-mimetic cellular model, we first screened the eight key metabolites of EF for their capacity to enhance NSC survival and proliferation under pathological Aβ stress. Building on these findings, we subsequently validated the therapeutic efficacy of the lead metabolite ICS II through *in vivo* assessment of hippocampal neurogenesis in adult APP/PS1 transgenic mice, with mechanistic investigations focusing on mitochondrial dynamics regulation.

## 2 Materials and methods

### 2.1 Materials

ICS II (Lot No. wkq19050410, purity ≥98%), ICA (Lot No. wkq21042608, purity ≥98%), Epimedin A (Lot No. wkq21060111, purity ≥98%), Epimedin B (Lot No. wkq21071613, purity ≥98%), Epimedin C (Lot No. wkq21041304, purity ≥98%), Icaritin (Lot No. wkq21042304, purity ≥98%), Anhydroicaritin (Lot No. wkq210430006, purity ≥98%) and ICS Ⅰ (Lot No. wkq21071908, purity ≥98%) were obtained from the Weikeqi Biotech (Chengdu, China), and the structures of the eight metabolites are shown in Supplementary Figure S1 in the supplementary materials. B27 supplement (17,054-044), 0.25% Trypsin (25,200,056) and Dulbecco’s Modified Eagle’s Medium/F12 (DMEM/F12) were purchased from Gibco (Rockville, MD). The epidermal growth factor (EGF, 315-09) and basic fibroblast growth factor (bFGF, 100-18B) were purchased from Pepro Tech (Suzhou, China). The P/S (J19007) was purchased from Hyclone (United States). The 5-bromo-2′-deoxyuridine (BrdU, MB3126-2) was purchased from the Meilun Biotech (Dalian, China). The Cell count kit-8 (CCK-8) and the lactate dehydrogenase (LDH) kit were purchased from Nanjing Jiancheng Biotechnology, Co. (Nanjing, China). The JC-1 kit was purchased from Biosynthesis Biotech (Beijing China). 5-ethynyl-2′-deoxyuridine (EdU, 61,135-33–9) was obtained from Sigma-Aldrich (Shanghai, China). DAPI (C1005) was purchased from Beyotime Biotechnology (Shanghai, China). The Rabbit anti Sex-determining region y-box 2 (Sox-2, 20118-1-AP) and rabbit anti-NeuN (26975-1-AP) were obtained from proteintech (Wuhan, China). The rabbit anti-Mitofusin 1 (Mfn 1, bs-0557R), rabbit anti-Mitofusin 2 (Mfn 2, bs-2988R), rabbit anti-Mitochondrial fission factor (Mff, bs-7628R), rabbit anti-Dynaminrelated protein 1 (Drp 1, bs-4100R), and rabbit anti-p-Ser616-Drp 1 (bs-12702R) were purchased from Biosynthesis Biotech (Beijing, China). Rabbit anti-GAPDH (WL01114) were purchased from Wanlei Biotech (Shenyang, China). The Goat anti-Rabbit IgG antibody conjugated with Alexa Fluor^®^ 488 (159082) was obtained from Jackson ImmunoResearch Inc. (West Grove, PA, United States).

### 2.2 Culture of NSCs and transfection with APPswe gene

Hippocampal NSCs were isolated from the hippocampus of neonatal C57BL/6 mice and cultured in proliferation medium consisting of DMEM/F12 supplemented with 20 ng/mL EGF, 20 ng/mL bFGF, 1% B27, 1% non-essential amino acids (NEAA), 1% GlutaMAX, and 0.5% penicillin/streptomycin, as previously described (Duan et al., 2015). To establish an AD cell model, hippocampal NSCs were transfected with either the APP-GFP lentiviral vector or a GFP lentiviral vector (negative control), as detailed in our prior studies (Xiao et al., 2020). The third passage of NSCs was used for subsequent experiments.

### 2.3 Cell viability and LDH assays

Cell viability was assessed using the CCK-8 assay. Briefly, a single-cell suspension was seeded into 96-well plates at a density of approximately 1 × 10^6^ cells/mL and cultured in proliferation medium for 24 h. Subsequently, the cells were treated with ICS II, ICA, Epimedin A, Epimedin B, Epimedin C, Icaritin, Anhydroicaritin, or ICS I at concentrations of 0, 0.25, 0.5, and 1 μM for an additional 24 h. After treatment, 10 μL of CCK-8 solution was added to each well, and the plates were incubated at 37°C for 4 h. Absorbance was measured at 450 nm using a microplate reader (MR-96, Mindray, Shenzhen, China).

The LDH assay was conducted to evaluate the cytoprotective effects of the aforementioned metabolites (ICS II, ICA, Epimedin A, Epimedin B, Epimedin C, Icaritin, Anhydroicaritin, ICS I) on APP-NSCs, following the manufacturer’s instructions. Absorbance at 450 nm was measured using the same microplate reader.

### 2.4 Neurosphere formation assay

Following dissociation of the neurospheres containing NSCs, cells were seeded at a density of 1 × 10^5^ cells/mL in a 24-well plate and treated with the selected active metabolite from Section 2.4, which demonstrated the most effective anti-cell damage properties. The cells were cultured under proliferative conditions, with media changes every 2 days. On day 7, neurosphere morphology and quantity were assessed using a microscope, and images were captured. The number of neurospheres was quantified using ImageJ software, and their diameters were measured.

### 2.5 BrdU incorporation assay

Single-cell suspension of NSCs was plated into a 96-well plate at a density of 1 × 10^6^ cells/mL and cultured in proliferation medium, with or without ICS II (1 μM), for 24 h. After 24 h, all cells were labeled with BrdU (20 μM) for 12 h, followed by fixation with 4% paraformaldehyde. The cells were then processed for immunofluorescent analysis as described by Shashi Kant (Shashi Kant et al., 2015).

### 2.6 JC-1 staining

GFP-NSCs and APP-NSCs cells in the logarithmic growth phase were divided into four groups: GFP-NSCs + PBS, GFP-NSCs + ICS II, APP-NSCs + PBS, and APP-NSCs + ICS II. Cells were seeded at a density of 5 × 10^3^ cells/well in a 96-well plate and cultured for 24 h. After incubation, the culture medium was removed, and equal volumes of H-DMEM and JC-1 staining solution were added. The plate was gently mixed and incubated in the dark at 37°C for 20 min. During the incubation, the 5× JC-1 staining buffer was diluted with four volumes of ddH_2_O and kept on ice. After the incubation period, the staining solution was discarded, and the cells were washed twice with freshly prepared 1× JC-1 staining buffer. Finally, 100 µL of H-DMEM was added, and the cells were observed under a microscope.

### 2.7 Reactive oxygen species (ROS) assay

The levels of ROS in NSCs and brain tissues were measured using the DCFH-DA fluorescent probe (E004-one to one, Nanjing Jiancheng Bioengineering Institute, Nanjing, China), as described in a previous study (Wen et al., 2021). NSCs from different experimental groups were treated with DCFH-DA solution for 30 min, and then collected, washed three times with sterile PBS. Fluorescence intensity was measured using an automated microplate reader.

### 2.8 Adenosine 5′-triphosphate (ATP) assay

The ATP levels in NSCs and brain tissues were measured using a firefly luciferase-based assay (S0027, Beyotime Biotechnology, Shanghai, China), as described in the previous study (Liu et al., 2021). NSCs cultured *in vitro* or brain tissues collected from mice across different groups were lysed, and the resulting supernatant was used to detect ATP levels according to the manufacturer’s instructions.

### 2.9 Animals and drug administration

Male APP/PS1 mice were purchased from Jiangsu Huachuang Sino Pharmaceutical Technology Co., Ltd. (SCXK [Su] 2020-0000), and male C57BL/6J mice were obtained from Liaoning Changsheng Biotechnology Co., Ltd. (SCXK [Liao] 2020-0001). All mice were housed in transparent brown plastic cages within a controlled environment (temperature: 25°C ± 1°C, humidity: 65% ± 5%, and a 12-hour light/dark cycle) with *ad libitum* access to food and water. All experiments were approved by the Institutional Animal Care and Use Committee (IACUC) of Liaoning University of Traditional Chinese Medicine (Approval No. SYXK [Liao] 2021-0001) and were conducted in accordance with the ARRIVE guidelines.

ICS II was prepared in a 0.5% sodium carboxymethyl cellulose (CMC-Na) aqueous solution. EdU (CAS 61135-33–9, Sigma-Aldrich, Shanghai, China) was dissolved in normal saline, while Rotenone (Rot, Aladdin, China, F2122218) was dissolved in a minimal volume of dimethyl sulfoxide (DMSO) and then diluted with sunflower seed oil. Six-month-old male APP/PS1 mice, weighing approximately 30 g, were used as the AD model, with wild-type C57BL/6J littermates serving as normal controls. APP/PS1 mice were randomly divided into five groups: Model, ICS II, Rotenone (Rot), ICS II + Rot, and Normal Control. The ICS II group received 10 mg/kg ICS II via oral gavage (i.g.) according to our previous study (Xiao et al., 2022), the Rot group was administered 2.5 mg/kg Rot via intraperitoneal injection (i.p.), and the ICS II + Rot group received both treatments (10 mg/kg ICS II via i.g. and 2.5 mg/kg Rot via i.p.). An additional ten C57BL/6J mice were included as normal controls (n = 10 per group). Mice in the Model, Control, and Rot groups were administered an equivalent volume of CMC-Na daily via oral gavage for 49 consecutive days.

After 4 weeks of treatment, the animals were intraperitoneally injected with EdU (100 mg/kg) to label proliferating cells in the hippocampus (Fatt et al., 2015). Following this, treatment continued for another 3 weeks with their respective drug regimens. At the end of the 49-day treatment period, the mice were euthanized, and brains were collected for immunofluorescence (IF) staining and Western blot analysis.

### 2.10 Nest-building test

The nest-building test was used to evaluate the self-care capacity of the mice, as previously described (Torres-Lista and Giménez-Llort, 2013; Xiao et al., 2022). The test was conducted on day 21 of drug administration, with assessments performed once daily for three consecutive days. Mice were housed individually in cages with corn cob bedding and provided with two 5 cm × 5 cm square sheets of sanitary paper (10 sheets per stack) at the feeding port. Nest-building behavior was scored at 0 h, 24 h, 36 h, and 72 h using a five-point scale. The scoring criteria were as follows: one point for no noticeable interaction or tearing of the paper; two points for partial tearing; three points for most of the paper torn, but not forming a distinct nest shape; four points for the paper torn into pieces and arranged into a relatively flat nest-like structure; and five points for the paper torn into pieces and built into a complete or nearly complete nest-like structure.

### 2.11 Morris water maze (MWM) test

The MWM test was used to evaluate the learning and memory abilities of the mice, as previously described. After completing the nest-building test, the MWM was conducted with daily assessments over six consecutive days. The test consisted of two phases: training trials and a probe trial. During the training phase, a platform was fixed in a specific location within the pool, and mice underwent two trials per day for five consecutive days. The time spent and the swimming distance to reach the escape platform were recorded to assess spatial learning ability. In the probe trial, the platform was removed, and the time spent in the target quadrant, as well as the number of crossings over the platform’s original location, were measured to evaluate spatial memory (n = 10).

### 2.12 H&E staining and Nissl staining

H&E Staining: Three mice per group were deeply anesthetized, and their brains were harvested and fixed in 4% formaldehyde solution for 24 h. After dehydration and clarification, the brains were embedded in paraffin, sectioned, and deparaffinized in xylene. The sections were then dehydrated through a graded ethanol series (100%, 95%, 80%, and 70%). Following this, the sections were stained with hematoxylin for 7 min, differentiated in 1% hydrochloric acid-alcohol for 10 s, and stained with eosin for 2 min. After dehydration, the sections were cleared in xylene and mounted with neutral resin. Pathological changes in the cortex and hippocampus were observed under a microscope.

Nissl Staining: Paraffin-embedded brain sections were deparaffinized and incubated in toluidine blue staining solution at 50°C in the dark for 7 min. The reaction was stopped when Nissl bodies became clearly visible. The sections were then rinsed twice in distilled water for 20 s each, dehydrated, cleared in xylene, and mounted with neutral resin. Alterations in the cortex and the number of Nissl bodies were observed under a microscope.

### 2.13 Immunofluorescence staining (IF) and EdU staining

Brain tissue sections from each mouse group were deparaffinized through a graded ethanol series and subjected to antigen retrieval in sodium citrate buffer. The sections were incubated with 2 mg/mL glycine for 10 min, followed by permeabilization with 0.5% Triton X-100 solution at room temperature for 10 min. Apollo staining solution was applied and incubated at room temperature for 30 min. After discarding the staining solution, sections were further permeabilized twice with 0.5% Triton X-100 for 10 min each. The sections were then washed with methanol for 5 min and blocked with 5% BSA solution at room temperature for 30 min. Primary antibodies (Sox-2: 1:150, NeuN: 1:200) were added and incubated overnight at 4°C. The following day, after washing with PBS three times, the sections were incubated with 488-labeled goat anti-rabbit secondary antibodies (1:100) at room temperature in the dark for 2 h. After three PBS washes, the sections were counterstained with DAPI-containing fluorescence quenching mounting medium, cover-slipped, and observed and photographed under an inverted fluorescence microscope. EdU staining was performed using the Cell-Light Apollo 567 Stain Kit (#C10371-1, Guangzhou RiboBio, Guangzhou, China) according to the manufacturer’s protocol.

### 2.14 Transmission electron microscopy (TEM) test

Three mice from each group were deeply anesthetized, and their brains were harvested to isolate hippocampal tissue from the ischemic side. The hippocampal CA1 region was dissected into 1 mm × 1 mm × 1 mm tissue blocks, which were fixed in electron microscopy fixative at 4°C for 24 h. After dehydration through a graded ethanol series and acetone, the samples were infiltrated, embedded, and sectioned into 70 nm ultrathin slices. The sections were double-stained with uranyl acetate and lead citrate before being examined under a TEM, and images were captured for analysis.

### 2.15 Western blot assays

Three mice from each group were deeply anesthetized, and the ischemic brain tissue was harvested and immediately frozen in liquid nitrogen. The tissue was then pulverized into a fine powder, and total protein was extracted using RIPA lysis buffer supplemented with 1% phosphatase inhibitor and 1% protease inhibitor. Protein concentrations were determined using the BCA method. Subsequently, proteins were separated by SDS-PAGE, transferred to PVDF membranes using wet transfer, and blocked at room temperature for 1.5 h. The membranes were incubated overnight at 4°C with primary antibodies against Mff (1:500), Drp1 (1:1,000), p-Drp1 (1:1,000), Mfn1 (1:500), Mfn2 (1:500), and GAPDH (1:1,000). The following day, the membranes were incubated with HRP-conjugated secondary antibodies for 2 h. Protein bands were visualized using a gel imaging system, and grayscale values were analyzed using ImageJ software.

### 2.16 Statistical analysis

Statistical analysis was performed using GraphPad Prism 9.4. The optical density in Western blot experiments was quantified with ImageJ software. For comparisons across multiple groups, one-way analysis of variance (ANOVA) followed by Dunnett’s post-hoc test was applied. For the escape latency data in MWM test, two-way ANOVA followed by Bonferroni post-hoc test was conducted. Results are presented as mean ± standard deviation (SD), with significance set at *P <* 0.05, indicating statistical significance.

## 3 Results

### 3.1 Protective effects of eight active metabolites from Epimedium Folium on APP-NSCs

**FIGURE 1:**
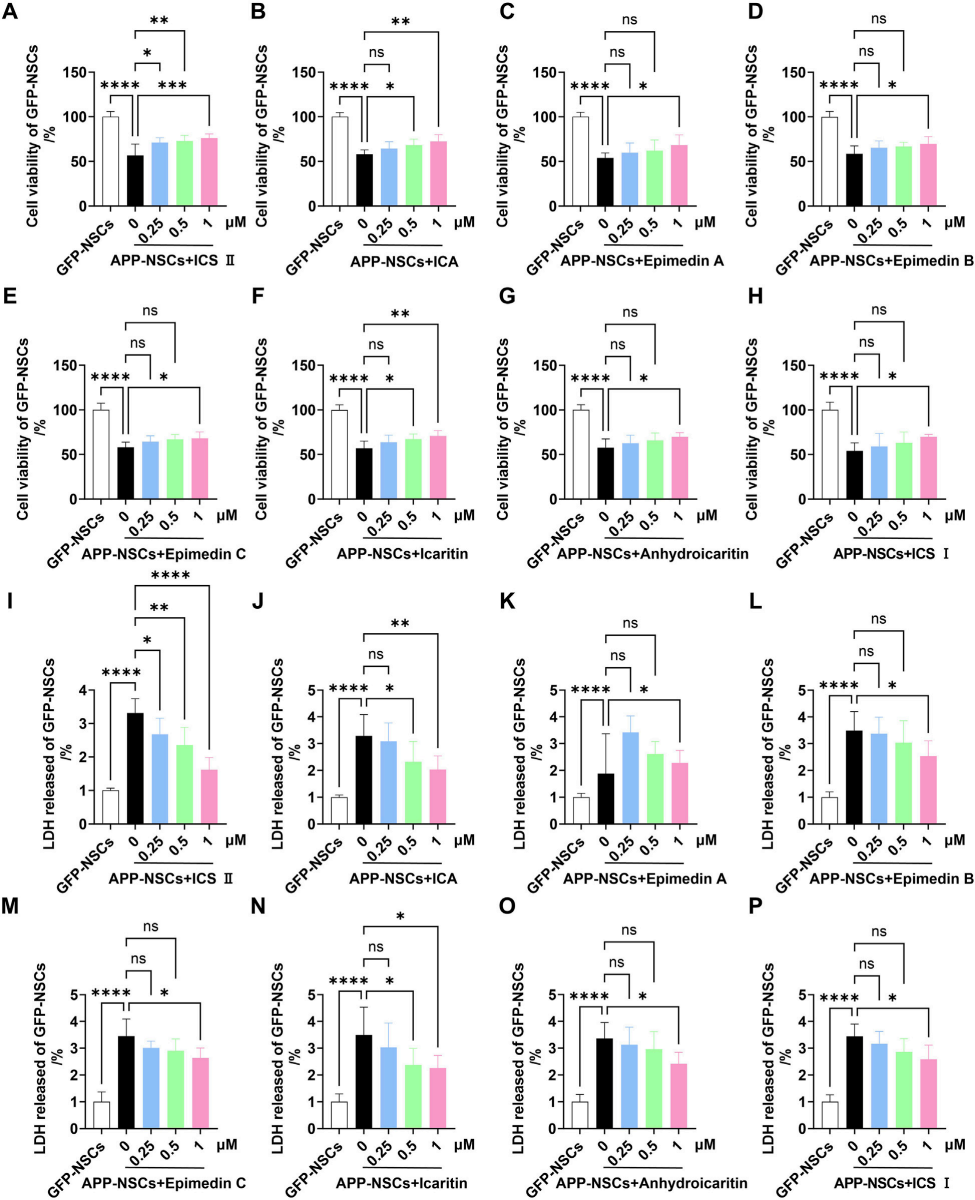
Protective effects of eight active metabolites from *Epimedium Folium* on APP-NSCs Monolayer APP-NSCs were cultured in proliferation medium for 24 h, followed by exposure to ICS II (0.25, 0.5, and 1 μM), ICA (0.25, 0.5, and 1 μM), Epimedin A (0.25, 0.5, and 1 μM), Epimedin B (0.25, 0.5, and 1 μM), Epimedin C (0.25, 0.5, and 1 μM), Icaritin (0.25, 0.5, and 1 μM), Anhydroicaritin (0.25, 0.5, and 1 μM), or ICS I (0.25, 0.5, and 1 μM) for another 24 h. Cell viability was assessed using the CCK-8 assay, and LDH leakage was measured by the LDH kit. **(A–H)** The viability of APP-NSCs exposed to the metabolites was measured by CCK-8 assay **(A)**: ICS II, **(B)** ICA, **(C)** Epimedin A, **(D)** Epimedin B, **(E)** Epimedin C, **(F)** Icaritin, **(G)** Anhydroicaritin, **(H)** ICS **(I)**. **(I–P)** LDH leakage in APP-NSCs exposed to the metabolites was assessed by LDH assay. Statistical significance: *P < 0.05; **P < 0.01; ***P < 0.001; ****P < 0.0001; ns: no significant difference. Data are presented as mean ± SD (n = 6/group). Data were analyzed by one-way ANOVA.

The CCK-8 assay revealed that APP-NSCs overexpressing the APP gene exhibited significantly reduced cell viability compared to GFP-NSCs (Figures 1A–H, ****P < 0.0001 vs GFP-NSCs, η^2^ = 0.819). Treatment with the eight active metabolites for 24 h partially restored cell viability, with ICS II, ICA, and Icaritin showing the most pronounced effects. These three metabolites demonstrated statistically significant improvements compared to the untreated APP-NSCs group (Figures 1A–H, ICS II: ***P < 0.001, η^2^ = 0.804; ICA: **P < 0.01, η^2^ = 0.859; Icaritin: **P < 0.01, η^2^ = 0.853 at 1 μM). Similarly, all eight metabolites reduced LDH release from APP-NSCs, with ICS II, ICA, and Icaritin showing the greatest reductions, which were also statistically significant compared to the APP-NSCs group (Figures 1I–P, ICS II: ****P < 0.0001, η^2^ = 0.827; ICA: **P < 0.01, η^2^ = 0.676; Icaritin: *P < 0.05, η^2^ = 0.623 at 1 μM). These findings indicate that ICS II, ICA, and Icaritin provide substantial protective effects for APP-NSCs. Consequently, these three metabolites were selected for subsequent NSC proliferation experiments. Notably, at the concentration of 0.25 μM, only ICS II demonstrated significant neuroprotective effects (cell viability: *P* < 0.05, η^2^ = 0.804; LDH release: *P* < 0.05, η^2^ = 0.827), whereas neither ICA nor Icaritin exhibited neuroprotective activity at the same concentration (cell viability: *P* > 0.05, η^2^ = 0.859; LDH release: *P* > 0.05, η^2^ = 0.676). These findings suggest that ICS II may represent the most potent neuroprotective metabolite in EF.

### 3.2 Effects of ICS II, ICA, and icaritin on the proliferation of APP-NSCs

**FIGURE 2:**
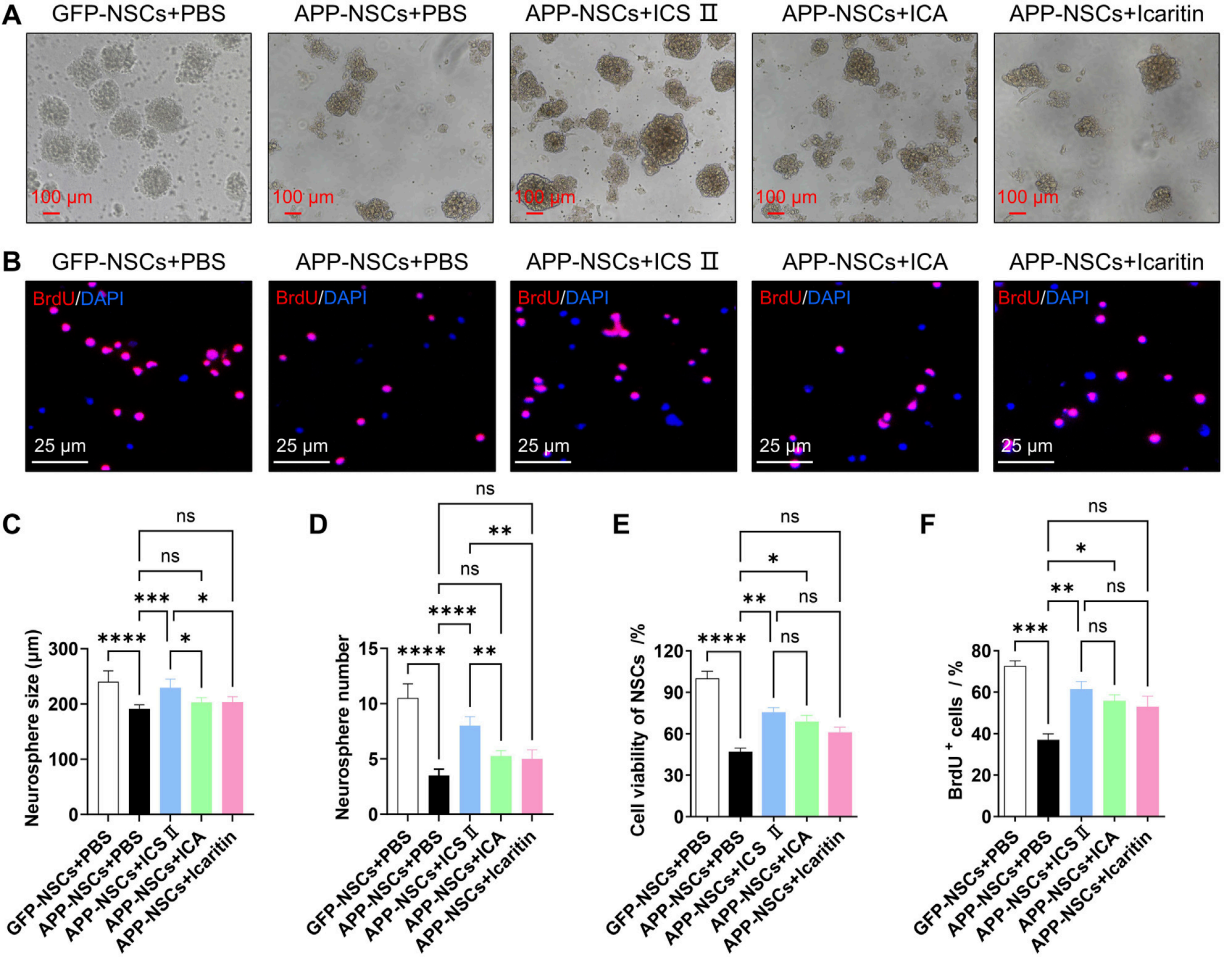
Effects of ICS II, ICA, and Icaritin on APP-NSCs proliferation APP-NSCs were cultured in proliferation medium and exposed to ICS II (1 μM), ICA (1 μM), or Icaritin (1 μM) for 7 days. Neurosphere images were captured, and their diameters were measured using ImageJ software. **(A)** Representative micrographs of neurospheres from each group. Scale bar = 100 μm. **(B)** Representative micrographs of BrdU^+^ proliferating cells. Scale bar = 25 μm. **(C)** Quantitative analysis of neurosphere diameters. **(D)** Quantification of neurosphere numbers. **(E)** Viability of BrdU^+^ proliferating cells. **(F)** Quantification of BrdU^+^ proliferating cells. Statistical significance: *P < 0.05; **P < 0.01; ***P < 0.001; ****P < 0.0001; ns: no significant difference. Data are presented as mean ± SD (n = 6/group). Data were analyzed by one-way ANOVA.

As shown in Figure 2, the APP-NSCs + PBS group exhibited significantly smaller neurosphere diameters and fewer neurospheres compared to the GFP-NSCs + PBS group (Figures 2A,C,D, ****P < 0.0001, C: η^2^ = 0.696, D: η^2^ = 0.920). Treatment with 1 μM ICS II significantly increased both the neurosphere diameter and number in APP-NSCs (Figures 2A,C,D, ****P < 0.0001, ***P < 0.001, C: η^2^ = 0.696, D: η^2^ = 0.920 vs APP-NSCs + PBS), while ICA and Icaritin exhibited no improvement in the neurosphere diameter or number of APP-NSCs (Figures 2A,C,D, *P* > 0.05, C: η^2^ = 0.696, D: η^2^ = 0.920).

Similarly, the proportion of BrdU-positive cells in the APP-NSCs + PBS group was markedly reduced compared to the GFP-NSCs + PBS group (Figures 2B,E,F, ****P < 0.0001, ***P < 0.001, E: η^2^ = 0.907, F: η^2^ = 0.845). Treatment with 1 μM ICS II or ICA significantly increased the proportion of BrdU-positive cells (Figures 2B,E,F, ICS II: **P < 0.01 vs APP-NSCs + PBS, ICA: *P < 0.05 vs APP-NSCs + PBS, E: η^2^ = 0.907, F: η^2^ = 0.845). Combined with the results of CCK-8 and LDH leakage in the 3.1 section, ICS II was determined as the most potential agent for subsequent experimental studies.

### 3.3 Protective effects of ICS II on mitochondria of APP-NSCs

**FIGURE 3:**
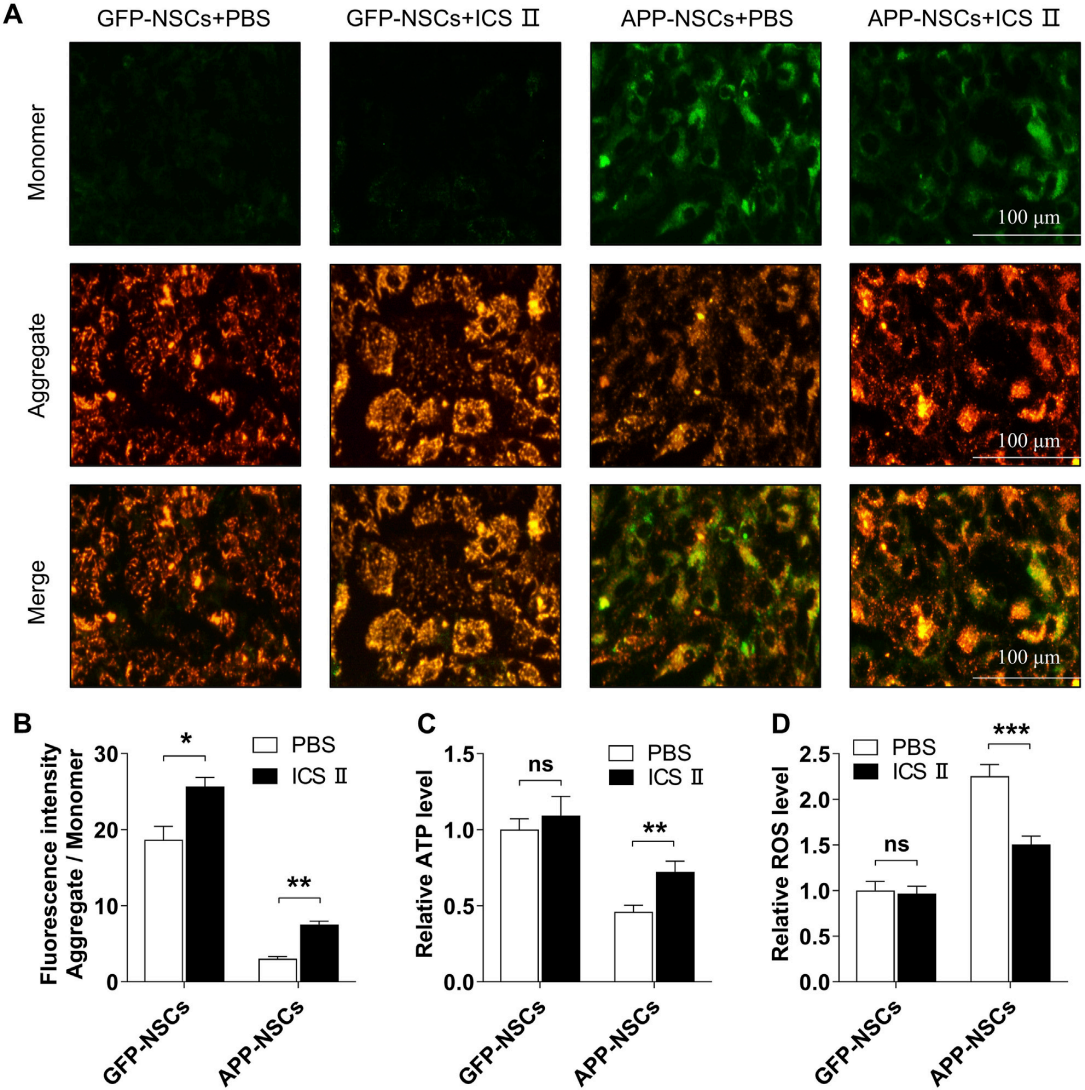
Effects of ICS II on mitochondrial function in APP-NSCs **(A)** Representative image of JC-1 staining; **(B)** Ratio of fluorescence intensity (Aggregate/Monomer); **(C)** ATP relative level. **(D)** ROS relative level. Statistical significance: *P < 0.05, **P < 0.01, ***P < 0.001; ns: no significant difference. Data are presented as mean ± SD (n = 6/group). Data were analyzed by T-test.

As illustrated in Figure 3, the APP-NSCs group showed a significant reduction in mitochondrial membrane potential compared to the GFP-NSCs group, as evidenced by decreased JC-1 aggregates and increased JC-1 monomers (Figure 3A). Treatment with 1 μM ICS II restored mitochondrial membrane potential by increasing the JC-1 aggregate-to-monomer fluorescence intensity ratio (Figure 3B, GFP-NSCs + ICS II: *P < 0.05 vs GFP-NSCs + PBS group, η^2^ = 0.729; APP-NSCs + ICS II: **P < 0.01 vs APP-NSCs + PBS group, η^2^ = 0.946). Furthermore, treatment with 1 μM ICS II significantly improved the ATP level (Figure 3C **P < 0.01, η^2^ = 0.509), and suppressed the ROS level (Figure 3D ***P < 0.001, η^2^ = 0.696) compared with that in the APP-NSCs + PBS group. These results demonstrate that 1 μM ICS II effectively alleviates mitochondrial damage.

### 3.4 ICS II enhances learning, memory, and self-care capacity in APP/PS1 mice

**FIGURE 4:**
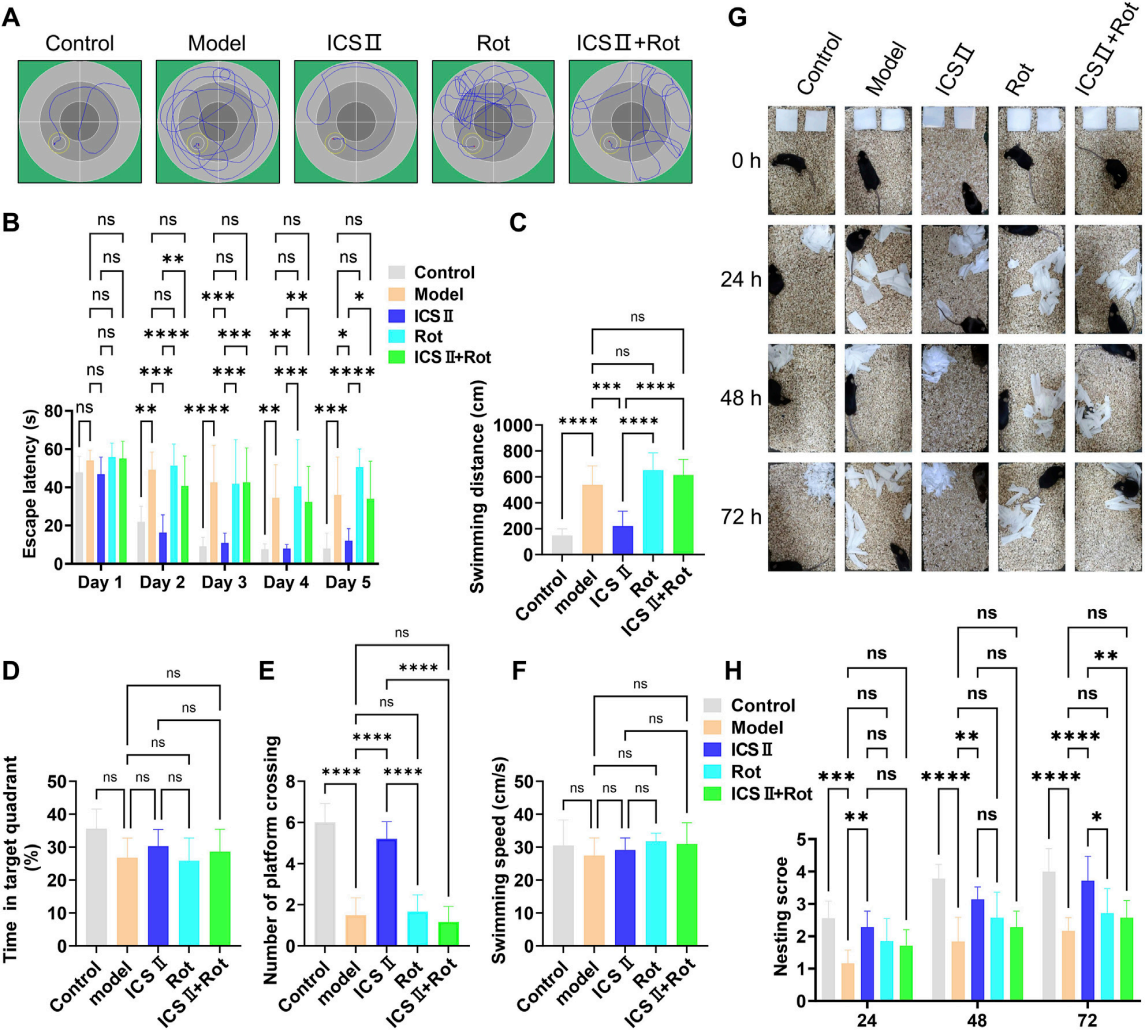
ICS II enhances learning, memory, and self-care capacity in APP/PS1 Mice **(A–F)** The Morris water maze (MWM) test was performed to assess cognitive function in each group of mice. **(A)** Typical swimming paths during the final training trial. **(B)** Escape latency during training trials. **(C)** Swimming distance before crossing the platform. **(D)** Percentage of time spent in the target quadrant. **(E)** Number of platform crossings. **(F)** Swimming speed during probe trials. **(G,H)** The nest building test was conducted following the MWM test to assess daily living activities. **(G)** Representative images of nests at 0, 24, 28, and 72 h. **(H)** Nesting score for each group. Data are presented as mean ± SD (n = 10 mice/group). Statistical significance: *P < 0.05; **P < 0.01; ***P < 0.001; ****P < 0.0001. Data were analyzed by repeated-measures two-way ANOVA with Bonferroni post-hoc test.

The MWM test was conducted to evaluate learning and memory abilities across different mouse groups. The typical swimming paths of each group are shown in Figure 4A. During training, escape latency gradually decreased in all groups, but the reduction was notably less pronounced in the Model group. From the second training day onward, the Model group demonstrated significantly prolonged escape latencies (Figure 4B, Day 2: **P < 0.01, Day 3: ****P < 0.0001, Day 4: **P < 0.01, Day 5: ***P < 0.001 vs Control group), increased swimming distances (Figure 4C, ****P < 0.0001 vs Control group, η^2^ = 0.797), and fewer platform crossings (Figure 4E, ****P < 0.0001 vs Control group, η^2^ = 0.883), indicating cognitive deficits in APP/PS1 mice. Treatment with ICS II significantly shortened escape latency (Figure 4B, Day 2: ***P < 0.001, Day 3: ***P < 0.001, Day 4: **P < 0.01, Day 5: *P < 0.05 vs Model group), reduced swimming distance (Figure 4C, ***P < 0.001 vs Model group, η^2^ = 0.797), and enhanced platform crossings (Figure 4E, ****P < 0.0001 vs Model group, η^2^ = 0.883), indicating that ICS II effectively improved learning and memory in APP/PS1 mice. However, these improvements were absent in the Rot and ICS II + Rot groups, with no significant differences compared to the Model group (^ns^ *P* > 0.05, Figure 4C: η^2^ = 0.797; Figure 4D: η^2^ = 0.294; Figure 4E: η^2^ = 0.883; Figure 4F: η^2^ = 0.650). This suggests that the cognitive benefits of ICS II depend on intact mitochondrial function.

The nesting experiment was used to assess the preserved executive function and self-care capacity. The Model group failed to complete nest-building within 72 h (Figures 4G,H, 24 h: ****P < 0.0001, 48 h: ***P < 0.001, 72 h: ***P < 0.001 vs Control group). In contrast, ICS II-treated mice exhibited nesting behavior within 24 h and formed relatively complete nests by 72 h, shredding paper into fragments to build the structure (Figure 4G). The nesting scores of the ICS II group were significantly higher than those of the Model group (Figure 4H, 24 h: **P < 0.01, 48 h: **P < 0.01, 72 h: ****P < 0.0001). However, co-treatment with Rot significantly reduced nesting scores (Figure 4H, 72 h: **P < 0.01 ICS II + Rot vs ICS II group). These results indicate that ICS II enhances the preserved executive function and self-care capacity of APP/PS1 mice, but this effect is hindered by Rot-induced mitochondrial dysfunction.

### 3.5 ICS II mitigates neuronal pathological damage in APP/PS1 mice

**FIGURE 5:**
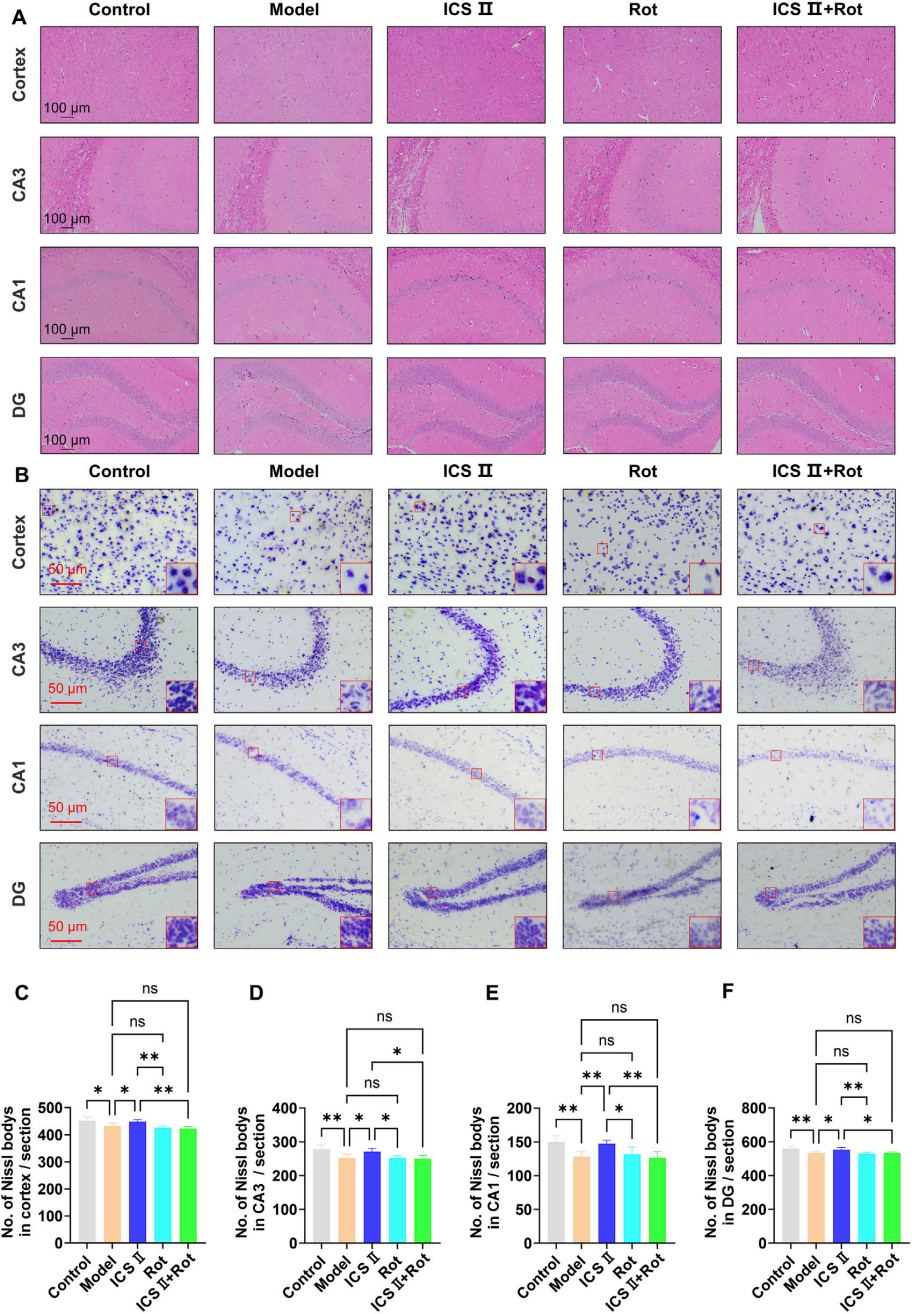
ICS II mitigates neuronal pathological damage in APP/PS1 Mice **(A)** Representative images of H&E staining (scale bar = 100 μm). **(B)** Representative images of Nissl staining in the cortex, hippocampal CA3, CA1, and DG regions (scale bar = 50 μm). **(C–F)** Quantitative analysis of Nissl bodies in the cortex **(C)**, hippocampal CA3 **(D)**, CA1 **(E)**, and DG **(F)** regions. Statistical significance: *P < 0.05; **P < 0.01; ns: no significant difference. Data are presented as mean ± SD (n = 3 mice/group). Data were analyzed by one-way ANOVA.

H&E staining revealed that neurons in the cortex and hippocampus of the Model group were loosely arranged, displayed irregular morphology, and exhibited hyperchromatic, shrunken nuclei (Figure 5A). In contrast, ICS II treatment improved neuronal organization, resulting in clearer layering, uniform cytoplasmic and nuclear staining, and a significant reduction in nuclear condensation (Figure 5A). Consistently, Nissl staining showed a marked reduction in the number of Nissl bodies in the cortex (Figures 5B,C, *P < 0.05 vs Control group, η^2^ = 0.638), hippocampal CA3 (Figures 5B,D, **P < 0.01 vs Control group, η^2^ = 0.611), CA1 (Figures 5B,E, **P < 0.01 vs Control group, η^2^ = 0.605) and dentate gyrus (DG) regions of the Model group (Figures 5B,F, **P < 0.01 vs Control group, η^2^ = 0.628). Treatment with ICS II significantly increased the number of Nissl bodies compared to the Model group, partially ameliorating neuronal damage (Figures 5B–F, Cortex: *P < 0.05, η^2^ = 0.638; CA3: *P < 0.05, η^2^ = 0.611; CA1: **P < 0.01, η^2^ = 0.605; DG: *P < 0.05, η^2^ = 0.628). However, neuronal damage remained unaltered in the Rot and Rot + ICS II groups compared to the Model group (Figures 5B–F, ^ns^ *P<*0.05). These results indicate that ICS II effectively alleviates hippocampal neuronal pathological damage in APP/PS1 mice, but its neuroprotective effects are blocked by Rot-induced mitochondrial dysfunction.

### 3.6 ICS II promotes hippocampal NSCs proliferation in APP/PS1 mice

**FIGURE 6:**
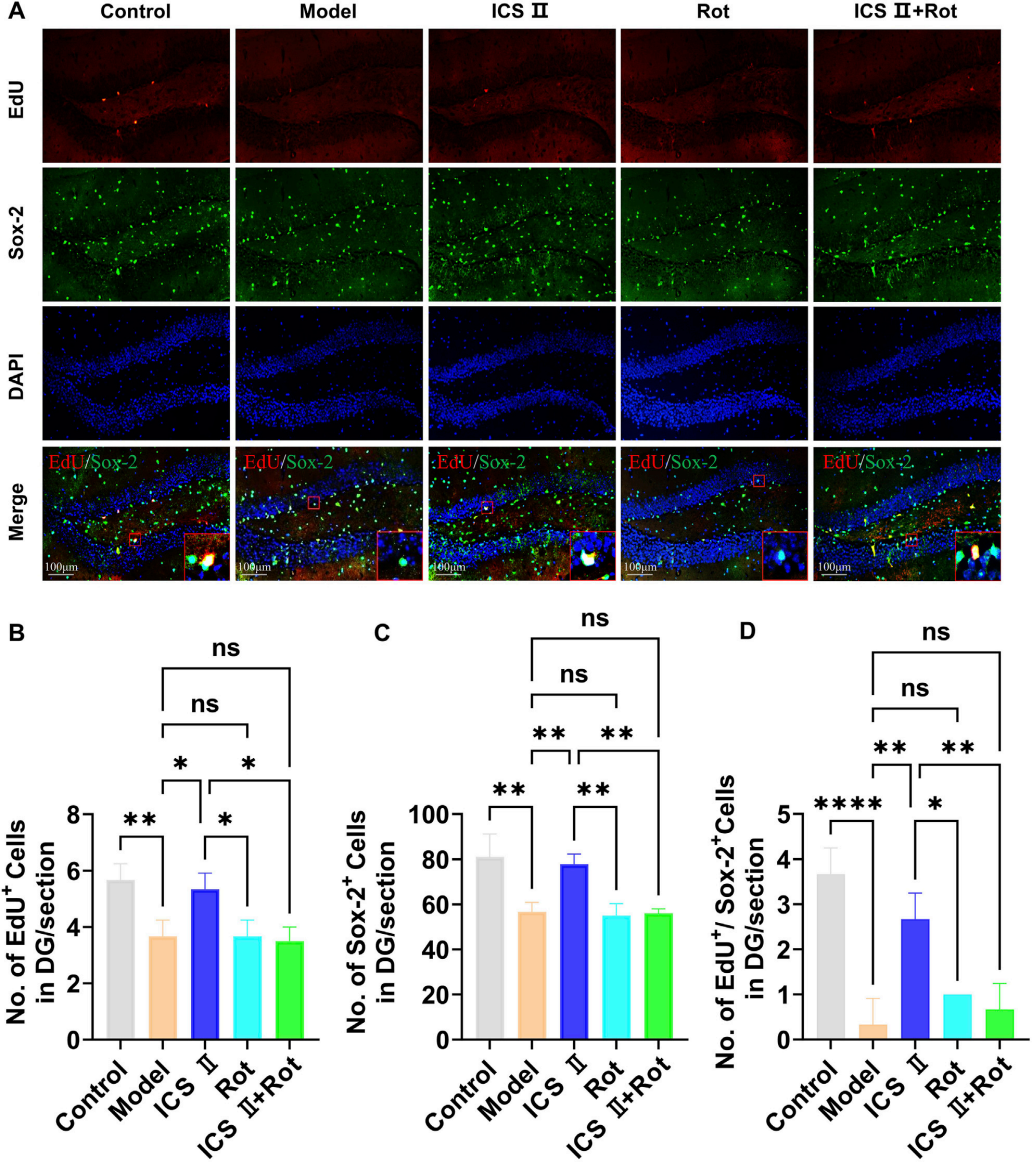
ICS II promotes hippocampal NSC proliferation in APP/PS1 Mice **(A)** Representative images of Sox-2 expression in the hippocampal DG region. Scale bar = 100 μm. **(B)** Quantification of EdU^+^ cells. **(C)** Quantification of Sox-2^+^ cells. **(D)** Quantification of EdU^+^/NeuN^+^ cells. Statistical significance: *P < 0.05; **P < 0.01; ****P < 0.0001; ns: no significant difference. Data are presented as mean ± SD (n = 3 mice/group). Data were analyzed by one-way ANOVA.

EdU is a thymidine analog that can integrate into replicating DNA molecules during cell proliferation, thereby marking proliferating cells. Sox-2 is a specific marker for NSCs. In this study, we employed EdU^+^/Sox-2^+^ immunofluorescence double staining to evaluate NSCs proliferation. As shown in Figure 6, the number of EdU^+^ positive cells (Figures 6A,B, **P < 0.01 vs Control group, η^2^ = 0.805), Sox-2^+^ positive cells (Figures 6A,C, **P < 0.01 vs Control group, η^2^ = 0.852), and EdU^+^/Sox-2^+^ double-positive cells (Figures 6A,D, ****P < 0.0001 vs Control group, η^2^ = 0.902) in the hippocampal DG region were significantly reduced in the Model group, indicating impaired hippocampal NSCs proliferation in APP/PS1 mice. However, following 4 weeks of ICS II treatment, the numbers of EdU^+^ positive cells (*P < 0.05, η^2^ = 0.805), Sox-2 positive cells (**P < 0.01, η^2^ = 0.852), and EdU^+^/Sox-2^+^ double-positive cells (**P < 0.01, η^2^ = 0.902) were significantly increased compared to Model group. Importantly, Rot aggravated the proliferation impairment of hippocampal neural stem cells in APP/PS1 mice and attenuated the proliferative effects of ICS II (EdU^+^/Sox-2^+^: **P < 0.01, ICS II + Rot vs ICS II; ^ns^ *P* > 0.05, ICS II + Rot vs Model group, η^2^ = 0.902). These findings suggest that ICS II promotes hippocampal neural stem cell proliferation in APP/PS1 mice, and this effect is dependent on mitochondrial function.

### 3.7 ICS II promotes hippocampal NSCs differentiation in APP/PS1 mice

**FIGURE 7:**
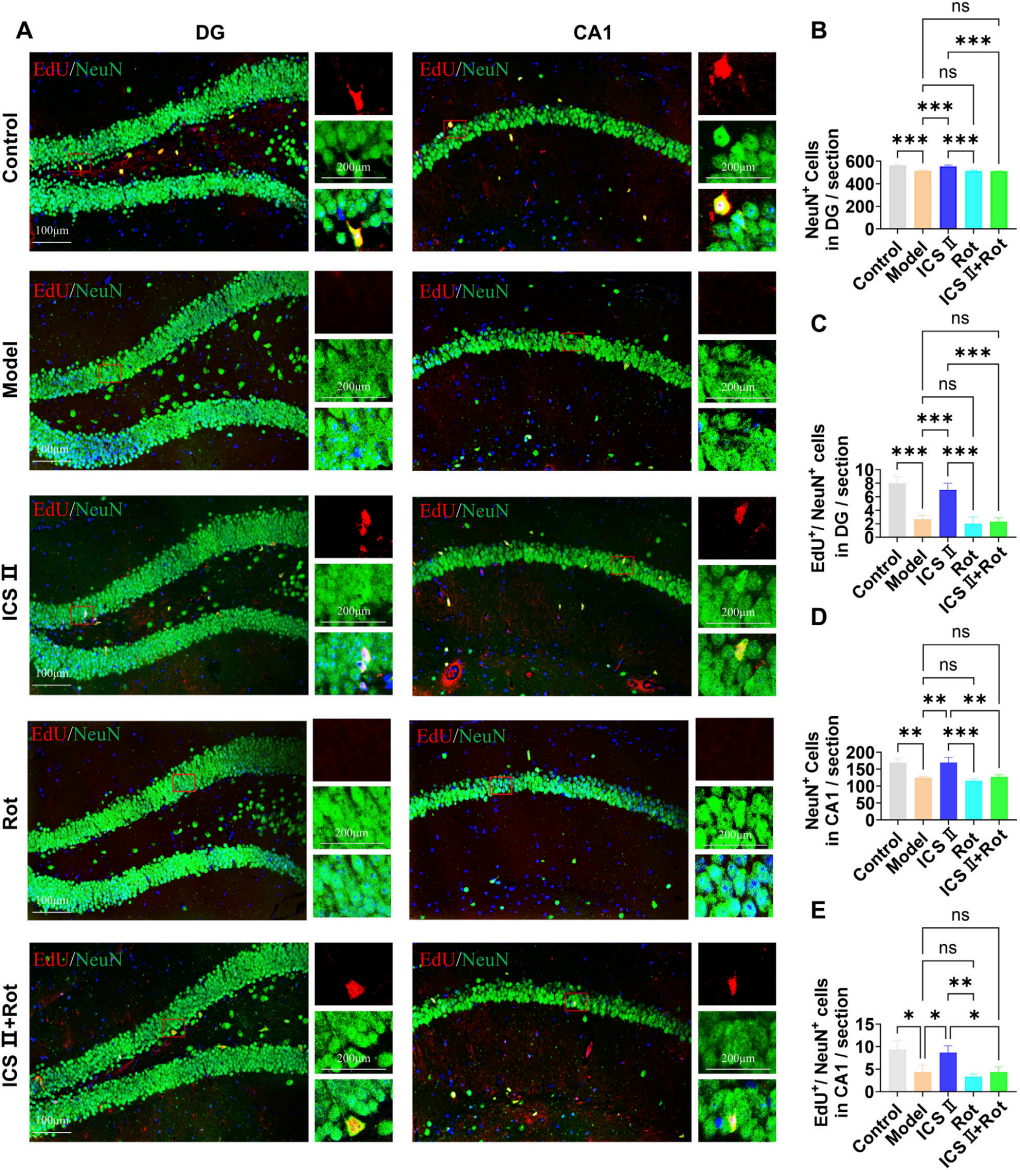
ICS II promotes hippocampal NSC differentiation in APP/PS1 Mice **(A)** Representative images of NeuN expression in the hippocampal DG and CA1 regions. Scale bar = 100 μm (original images), 200 μm (magnified images). **(B,D)** Quantification of NeuN^+^ cells in the hippocampal DG **(B)** and CA1 **(D)** regions. **(C,E)** Quantification of EdU^+^/NeuN^+^ cells in the hippocampal DG **(C)** and CA1 **(E)** regions. Statistical significance: *P < 0.05; **P < 0.01; ***P < 0.001; ****P < 0.0001; ns: no significant difference. Data are presented as mean ± SD (n = 3 mice/group). Data were analyzed by one-way ANOVA.

The differentiation of NSCs was assessed using EdU/NeuN immunofluorescence double staining. As shown in Figure 7, compared to the Control group, the number of NeuN-positive cells (CA1: **P < 0.01, η^2^ = 0.903; DG: ***P < 0.001, η^2^ = 0.928) and EdU^+^/NeuN^+^ double-positive cells in the hippocampal CA1(*P < 0.05, η^2^ = 0.813) and DG (***P < 0.001, η^2^ = 0.931) regions was significantly reduced in the Model group, indicating impaired differentiation of hippocampal NSCs in APP/PS1 mice. After 7 weeks of ICS II treatment, the numbers of NeuN positive cells (CA1: **P < 0.01, η^2^ = 0.903; DG: ***P < 0.001, η^2^ = 0.928 vs. Model group) and EdU^+^/NeuN^+^ double-positive cells (CA1: *P < 0.05, η^2^ = 0.813; DG: ***P < 0.001, η^2^ = 0.931 vs. Model group) were significantly increased, demonstrating that ICS II promotes the differentiation of hippocampal NSCs. Similar to the proliferation results, Rot further inhibited hippocampal neurogenesis in APP/PS1 mice and negated the effect of ICS II on increasing the number of EdU^+^/NeuN^+^ double-positive cells (CA1: *P < 0.05, ICS II + Rot vs ICS II; ^ns^ *P* > 0.05, ICS II + Rot vs Model group, η^2^ = 0.813; DG: ***P < 0.001, ICS II + Rot vs ICS II; ^ns^ *P* > 0.05, ICS II + Rot vs Model group, η^2^ = 0.931). These findings suggest that ICS II promotes the differentiation of hippocampal NSCs, and this effect is dependent on the normal function of mitochondria.

### 3.8 ICS II inhibits mitochondrial hyper-division in APP/PS1 mice

**FIGURE 8:**
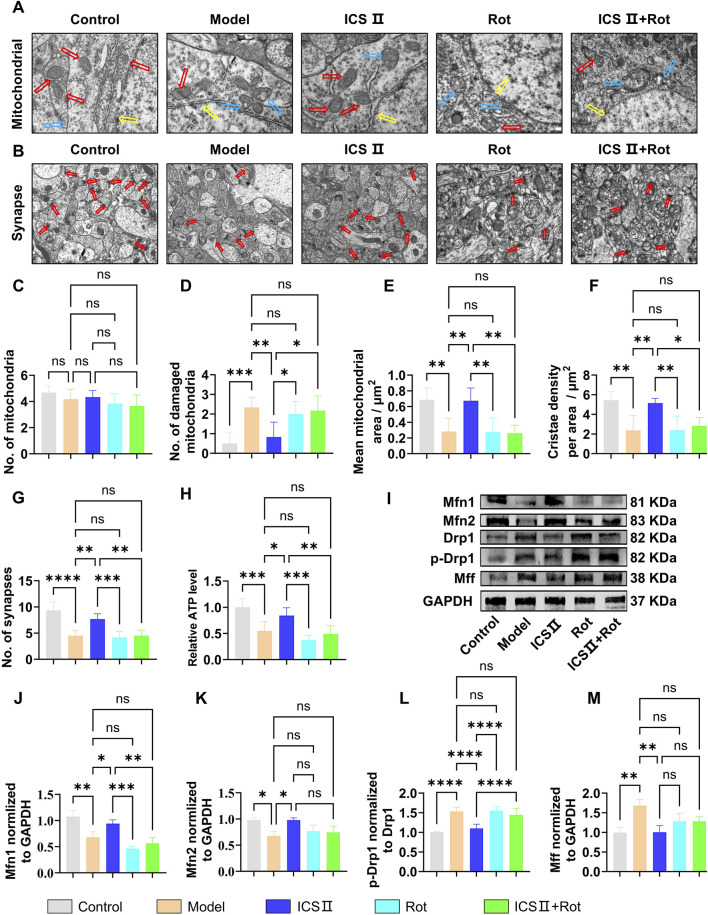
ICS II inhibits mitochondrial hyper-division in APP/PS1 Mice **(A)** Ultrastructural images of mitochondria (red arrow: healthy mitochondria, blue arrow: damaged mitochondria, yellow arrow: nuclear membrane) in neurons detected by TEM. Scale bar = 500 nm. **(B)** Ultrastructural images of synapses (red arrow: synapse) in neurons detected by TEM, Scale bar = 1 μm, (n = 3 mice/group). **(C)** Total number of mitochondria. **(D)** Number of damaged mitochondria. **(E)** Mean mitochondrial area as in **(A)**. **(F)** Mitochondria cristae density as in **(A)**. **(G)** Number of synapses as in **(A)**. **(H)** ATP relative level (n = 10 mice/group). **(I–M)** Changes in the expression levels of mitochondrial dynamics-related proteins (n = 3 mice/group). **(I)** Representative Western blot bands. **(J–M)** Quantitative analysis of Mfn1 **(J)**, Mfn2 **(K)**, p-Drp1/Drp1 **(L)**, and Mff **(M)** protein expression. Statistical significance: *P < 0.05; **P < 0.01; ***P < 0.001; ****P < 0.0001; ns: no significant difference. Data are presented as mean ± SD. Data were analyzed by one-way ANOVA followed by Bonferroni post-hoc test.

As shown in Figures 8A–G, neurons in the Model group displayed folded and ruptured nuclear membranes (Figure 8A, yellow arrow), increased number of damaged mitochondria (Figures 8A,D, blue arrow, ***P < 0.001, η^2^ = 0.616), decreased mitochondrial area (Figures 8A,E, **P < 0.01, η^2^ = 0.674) and cristae density (Figures 8A,F, **P < 0.01, η^2^ = 0.656), as well as reduced number of synapses (Figures 8B,G, ****P < 0.0001, η^2^ = 0.781) compared with Control group, indicating severe ultrastructural damage of neurons and mitochondria. Moreover, the ATP production was significantly decreased in Model group (***P < 0.001, η^2^ = 0.703). In contrast, ICS II treatment resulted in significant restoration of these mitochondrial damages, as evidenced by decreased number of damaged mitochondria (Figures 8A,D, **P < 0.01, η^2^ = 0.616), increased mitochondrial area (Figures 8A,E, **P < 0.01, η^2^ = 0.674) and cristae density (Figures 8A,F, **P < 0.01, η^2^ = 0.656), increased number of synapses (Figures 8B,G, **P < 0.01, η^2^ = 0.781), and higher level of ATP (*P < 0.001, η^2^ = 0.703) compared with Model group. Rot blocked the protective effects of ICS II on neuronal ultrastructure and mitochondria.

As shown in Figures 8F–J, the Model group exhibited significantly reduced expression of mitochondrial fusion proteins Mfn1 (Figures 8I,J, **P < 0.01, η^2^ = 0.898) and Mfn2 (Figures 8I,K, *P < 0.05, η^2^ = 0.756), while the expression of fission proteins p-Drp1/Drp1(Figures 8I,L, ****P < 0.0001, η^2^ = 0.833) and Mff (Figures 8I,M, **P < 0.01, η^2^ = 0.788) was significantly elevated compared to the Control group, suggesting abnormal mitochondrial dynamics in the hippocampus of APP/PS1 mice. After 7 weeks of ICS II treatment, the expression of mitochondrial fusion proteins Mfn1(Figures 8I,J, *P < 0.05 vs Model group, η^2^ = 0.898) and Mfn2 (Figures 8I,K, *P < 0.05 vs Model group, η^2^ = 0.756) was significantly increased, while the expression of mitochondrial fission proteins p-Drp1/Drp1(Figures 8I,L, ****P < 0.0001 vs Model group, η^2^ = 0.833) and Mff (Figures 8I,M, **P < 0.01 vs Model group, η^2^ = 0.788) was reduced. Simultaneous administration of Rot blocked ICS II’s effect in correcting the abnormal protein expression associated with mitochondrial dynamics. These results indicate that ICS II enhances mitochondrial fusion protein expression and reduces mitochondrial fission protein expression. The mitochondrial dysfunction induced by Rot blocks these effects.

## 4 Discussion

Neurogenesis is the process by which NSCs generate new neurons to restore existing neural circuits and repair damaged brain tissue. This process is essential for maintaining synaptic plasticity and ensuring proper central nervous system function. Extensive research has established that impairments in neurogenesis contribute to the pathogenesis and progression of AD (Disouky and Lazarov, 2021; Scopa et al., 2020; Young, 2020). In this study, we initially conducted *in vitro* experiments to assess the effects of eight active metabolites of EF on the survival and proliferation of APP-NSCs, identifying ICS II as the most effective protective metabolite. Subsequent animal experiments confirmed that ICS II promotes hippocampal neurogenesis and enhances cognitive function in AD mice. We demonstrated that ICS II stimulates hippocampal neurogenesis by regulating mitochondrial dynamics, thereby improving cognitive function in APP/PS1 mice.

EF is a traditional Chinese medicine used to tonify the kidneys, with multiple protective effects on the central nervous system. It contains various chemical metabolites, including flavonoids, polysaccharides, lignans, and alkaloids, among which flavonoids are the primary pharmacologically active metabolites. These include Epimedin A, Epimedin B, Epimedin C, ICA, ICS II, Icaritin, Anhydroicaritin, and ICS I (Li et al., 2020; Wang et al., 2016; Zhang L. B. et al., 2022; Zhuang et al., 2023). Epimedin A and Epimedin C have primarily been studied in the context of osteoporosis (Li et al., 2024; Xu et al., 2022). Epimedin B has been reported to counteract MPTP-induced dopaminergic neuronal damage and improve behavioral function in a Parkinson’s mouse model (Zhang M. et al., 2022). Icaritin exerts neuroprotective effects through estrogen-like receptor pathways, mitigating Aβ-induced neuronal damage (Wang et al., 2007), promoting the proliferation and differentiation of oligodendrocyte precursor cells, and improving cuprizone-induced demyelination (Yang et al., 2023). ICS II has been found to mediate signaling pathways, including BDNF/TrkB/CREB and Wnt/β-catenin, thereby improving cognitive function in various brain disease models (Xiao et al., 2022; Yin et al., 2018).

Mitochondria are double-membraned organelles that not only provide energy for cells but also play a crucial role in regulating biological processes such as signal transduction, nuclear gene transcription, and epigenetic modifications (Seo et al., 2018; Zhang et al., 2018). A 2019 review in Nature Reviews Neuroscience emphasized the critical role of mitochondria in regulating the proliferation and differentiation of NSCs, positioning them as key regulators of neurogenesis (Mireille et al., 2019). The activation, proliferation, and differentiation of NSCs require substantial energy and metabolic support. In resting NSCs, the mitochondrial network is fragmented, the cristae are underdeveloped, and mitochondrial function is immature, relying primarily on glycolysis for ATP production. Upon mitochondrial fusion and elongation, along with cristae maturation, mitochondrial DNA (mtDNA) copy numbers increase, and the energy supply shifts to oxidative phosphorylation, promoting the proliferation and differentiation of NSCs (Seo et al., 2018; Zhang et al., 2018). Studies have shown that knockout of mitochondrial fusion genes in NSCs impairs hippocampal NSC proliferation and differentiation, resulting in cognitive deficits in mice (Khacho et al., 2016). In AD, mitochondrial dynamics are disrupted, leading to excessive fragmentation of the mitochondrial network, reduced respiratory chain enzyme activity, decreased ATP production, increased mtDNA mutations, and elevated ROS levels, all of which exacerbate neurogenesis impairment (Monzio Compagnoni et al., 2020; Shevtsova et al., 2021; Wang et al., 2020). Additionally, studies have shown that inhibiting mitochondrial fission with Mdivi-1 promotes mitochondrial fusion, increases ATP levels, and enhances hippocampal neurogenesis in a Down syndrome mouse model (Valenti et al., 2017).

In the present study, we found that ICS II effectively enhanced mitochondrial membrane potential, increased ATP production, and reduced ROS levels in APP-NSCs (Figure 3), indicating promising mitochondrial protection. *In vivo* experiments demonstrated that continuous administration of 10 mg/kg ICS II for 4 weeks promoted the proliferation of hippocampal NSCs (EdU^+^/SOX-2^+^, Figure 6), while a 7-week treatment further enhanced their differentiation (EdU^+^/NeuN^+^, Figure 7), confirming the neurogenic effects of ICS II. Notably, ICS II improved ATP production, promoted the expression of mitochondrial fusion proteins Mfn1 and Mfn2, inhibited the expression of mitochondrial fission proteins p-Drp1/Drp1 and Mff, and alleviated mitochondrial ultrastructural damage in the brain of APP/PS1 mice (Figure 8).

To further investigate whether the neurogenesis-promoting effect of ICS II depends on the protection of mitochondrial structure and function, we administered Rot to disrupt the mitochondrial respiratory chain, thereby impairing mitochondrial structure and function, and assessed neurogenesis. The results showed that in the presence of Rot, the neurogenesis-promoting effect of ICS II was significantly diminished, with a marked difference compared to ICS II alone. These findings suggest that the neurogenesis-enhancing effect of ICS II is indeed dependent on the preservation of mitochondrial structure and function.

Recent research has identified several natural metabolites capable of modulating mitochondrial function. For instance, huperzine A enhances the activity of respiratory chain complexes, thereby reducing the accumulation of subcellular amyloid-beta (Aβ) (Yang et al., 2012). Ginsenoside Rg1 improves mitochondrial membrane potential and interconnectivity (Kwan et al., 2021). Salvianolic acid B mitigates mitochondrial stress and preserves mitochondrial bioenergetics against Aβ toxicity (He et al., 2018). In our study, ICS II treatment resulted in the suppression of Drp1 hyperactivation and Mff overexpression, alongside an increase in Mfn1 and Mfn2 levels (Figure 8), providing the first evidence that ICS II restores mitochondrial homeostasis by balancing fusion and fission proteins, a process crucial for NSC proliferation and differentiation. Unlike previous reports, we link the restoration of mitochondrial dynamics to enhanced neurogenesis in APP/PS1 mice, addressing a critical gap in understanding how ICS II alleviates the bioenergetic deficits underlying AD pathogenesis. Considering the complexity of AD-related mitochondrial dysfunction, ICS II’s potential to promote neurogenesis and protect mitochondrial function may offer synergistic benefits.

This study primarily focused on the pharmacodynamics and mechanisms of action of ICS II, and does not include pharmacokinetic data. Therefore, the findings from animal models may not fully reflect human responses, particularly regarding metabolic pathways and BBB permeability. Clinical trials are needed to validate ICS II’s safety and efficacy in humans. Additionally, long-term toxicity and off-target effects were not explored in this study, highlighting the need for further preclinical and clinical research.

In conclusion, integrated *in vitro* screening identified ICS II as one of the most effective metabolites of EF for promoting NSC proliferation. Subsequent *in vivo* studies demonstrated its ability to enhance hippocampal neurogenesis, primarily through the regulation of mitochondrial dynamics, highlighting its therapeutic potential for AD.

## Data Availability

The original contributions presented in the study are included in the article/Supplementary Material, further inquiries can be directed to the corresponding authors.

## Abbreviations

AD, Alzheimer’s disease; NSCs, neural stem cells; SGZ, subgranular zone; EF, Epimedii Folium; ICA, Icariin; ICS I, Icariside I, ICS II, Icariside II; NeuN, neuron-specific nuclear protein; APP-NSCs, neural stem cells overexpressing the APPswe mutant amyloid precursor protein; CCK-8, Cell Counting Kit-8; EGF, epidermal growth factor; bFGF, basic fibroblast growth factor, NEAA, non-essential amino acids; H&E, Hematoxylin-Eosin; DG, dentate gyrus; BSA, bovine serum albumin; SD, standard deviation; ANOVA, analysis of variance; TEM, transmission electron microscopy; BrdU, 5-bromo-2′-deoxyuridine; EdU, 5-ethynyl-2′-deoxyuridine; LDH, lactate dehydrogenase; Sox-2, sex-determining region; Mfn 1, mitofusin 1; Mfn 2, mitofusin 2; Mff, mitochondrial fission factor; Drp 1, dynaminrelated protein 1; Rot, rotenone; MWM, Morris water maze; mtDNA, mitochondrial DNA; ROS, reactive oxygen species; ATP, adenosine 5′-triphosphate.
