# Supplementary material for: The active metabolite of Epimedii Folium promotes hippocampal neurogenesis in APP/PS1 mice by alleviating mitochondrial dysfunction
Source: Front Pharmacol. 2025 Apr 25;16:1546256. doi: 10.3389/fphar.2025.1546256 (PMC12062837; doi:10.3389/fphar.2025.1546256)
Supplement: Supplementary file 1 [file DataSheet2.pdf]

产 品 分 析 报 告

Product Analysis Report

产品信息(Product information)

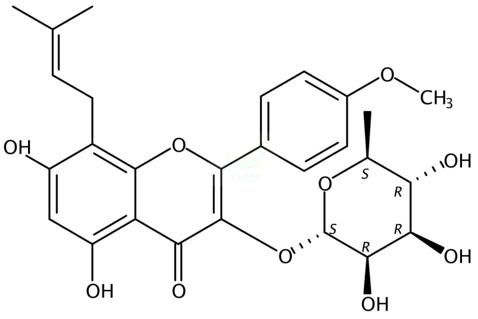

产品名称（Product Name）	
宝霍苷I（Baohuoside I）	
CAS号 （CAS No.）	113558-15-9
分子式（Mol. Formula）	C27H30O10
分子量（Mol. Weight）	514.53
批 号（Batch Number）	wkq19050410
报告日期（Report Date）	2019-05-04

报告分析（ Report analysis）

项目（Characters）	规定（Provisions）	结果（Results）
性状（Characteristics）	黄色粉末（Yellow powder）	符合（Conforms）
纯度Purity(HPLC)	≥98%	99.81%

审核人：　　　　负责人：

宝霍苷I 113558-15-9 检测条件

仪器：Waters HPLC;　　色谱柱：C18, 150×4.6mm,5μm;

流动相A：乙腈;　　流动相B：0.1%甲酸-水;

梯度洗脱：

时间（min）	A (%)	B (%)
0	90	10
20	10	90

流速：1.0 ml/min;　　柱温：25 ºC;　　波长：UV-269 nm;

进样浓度：0.5mg/ml;　　进样量：10μL;　　溶解方式：甲醇;

注意事项（Notes）：

使用范围：本品仅供实验、科研使用，不能用于人体医用或食用！

Scope of use: This product is only for experiment and scientific research, not for human medical use or consumption!

贮存方法：密封、干燥、避光、低温保存，即配即用。

Notes:Storage method: Store in sealed, dry, dark and low temperature conditions. Use immediately after configuration.

The URL：www.weikeqi-biotech.com　　Tel：0086-028-81700200　　4008005713

Office Address: Room 1630, 1631, 1632, Unit 1, Building 3, Qingyang Wanda, No. 978, Section 1, Riyue Avenue, Qingyang District, Chengdu

OLab Address：Chengdu Institute of Biology, Chinese Academy of Sciences,93 Keyuan South Road, Tianfu Life Science Park

宝霍苷I液相

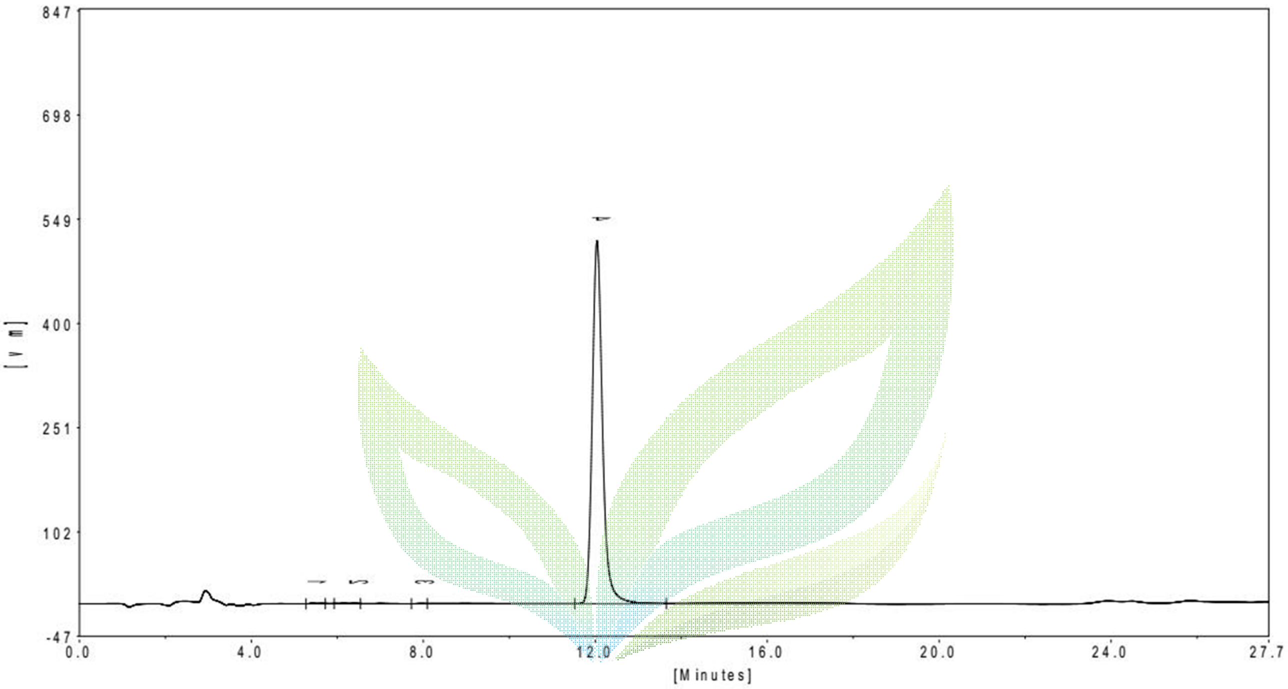

维克奇生物
WEI KE QI SHENG WU

#	组分名	保留时间(min)	峰高(mv)	峰面积(mv.sec)	面积百分比(%)
1	Unknown	5.42	0.61	6.16	0.0718
2	Unknown	6.40	0.54	7.33	0.0855
3	Unknown	7.94	0.22	2.08	0.0243
4	Icariside II	12.04	518.66	8563.73	99.8184
