# Supplementary material for: The active metabolite of Epimedii Folium promotes hippocampal neurogenesis in APP/PS1 mice by alleviating mitochondrial dysfunction
Source: Front Pharmacol. 2025 Apr 25;16:1546256. doi: 10.3389/fphar.2025.1546256 (PMC12062837; doi:10.3389/fphar.2025.1546256)
Supplement: Supplementary file 2 [file DataSheet1.docx]

Original images for checking

Purple arrows: target proteins

Red arrows: markers


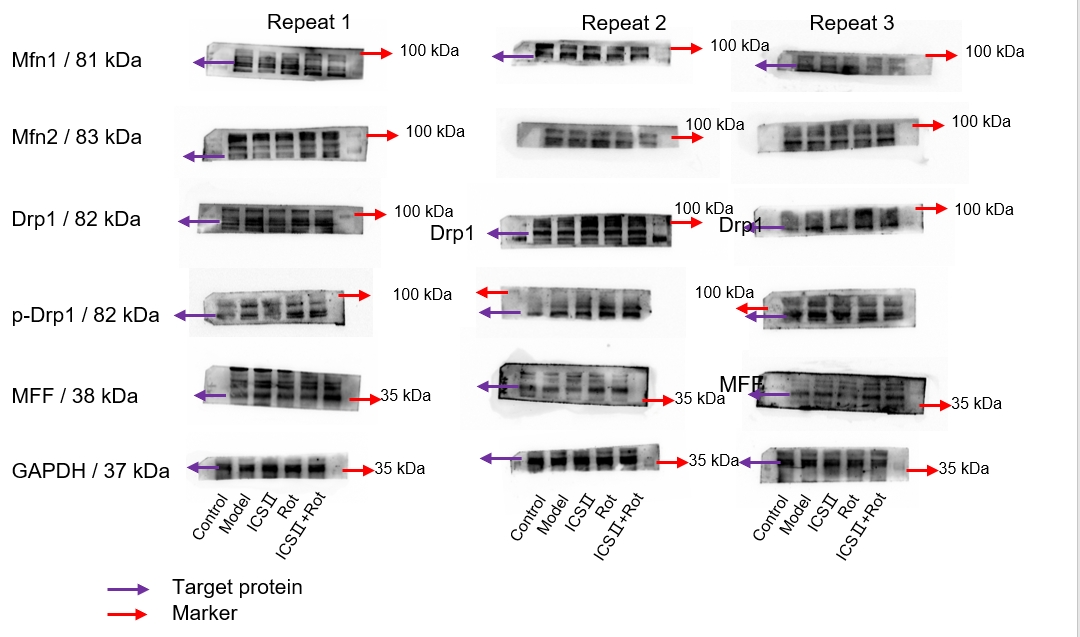


Figure S1 Structures of the 8 metabolites of Epimedii Folium.


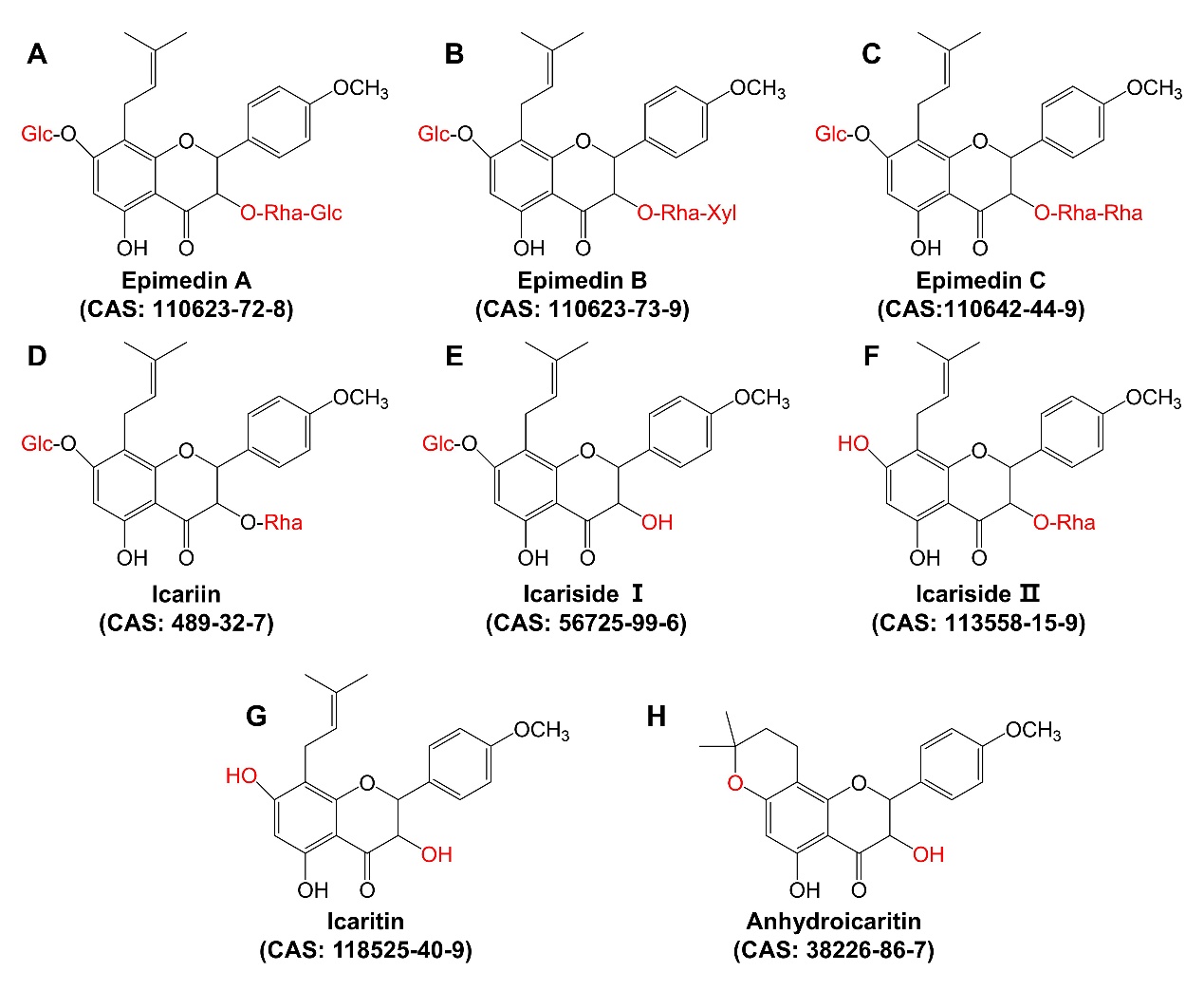


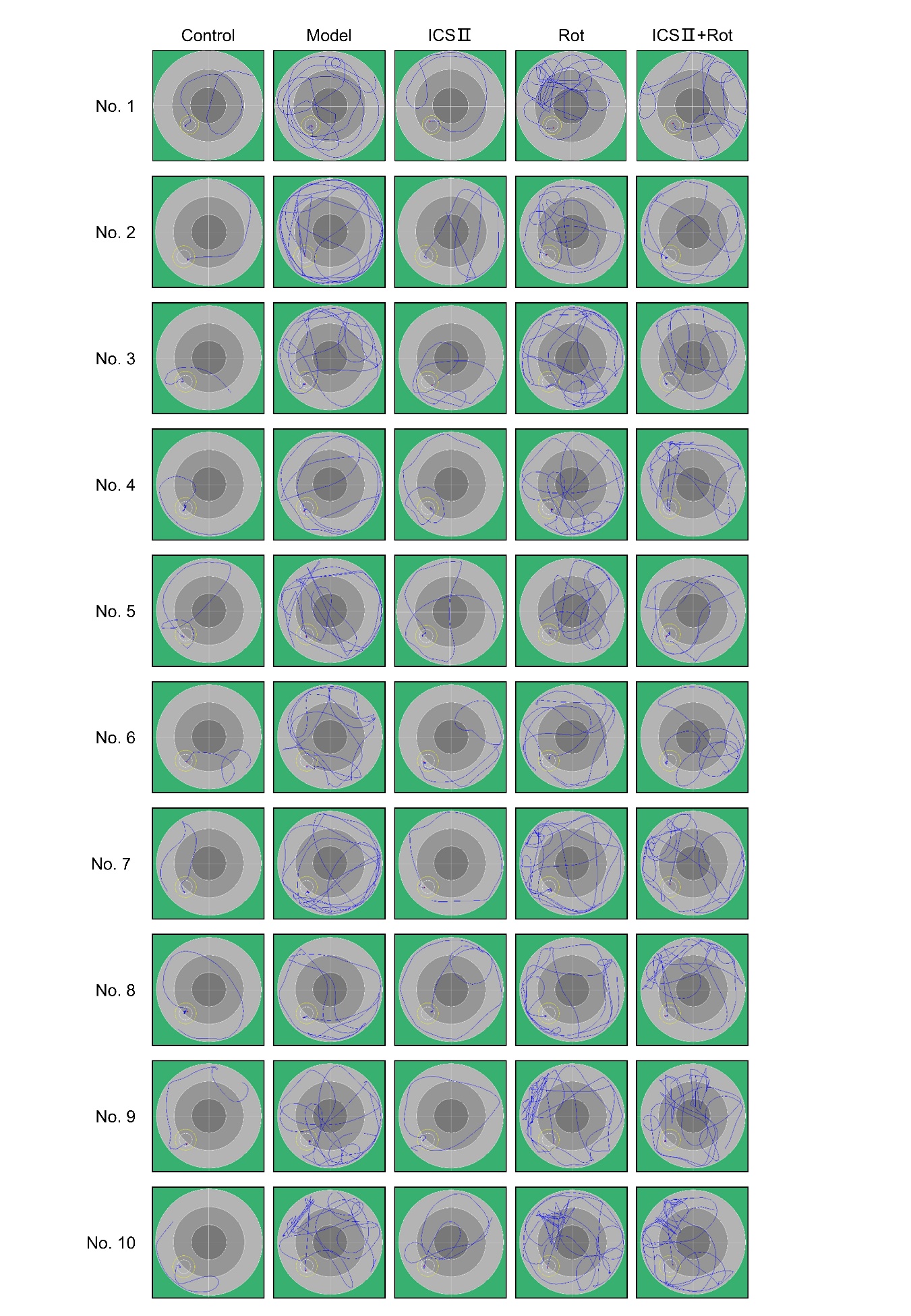
Figure S2 Swimming trajectory diagrams of mice in each group during the Morris Water Maze test. N=10 mice/group.
